# Computational Methods in Neuroengineering

**DOI:** 10.1155/2013/617347

**Published:** 2013-07-01

**Authors:** Chang-Hwan Im, Lei Ding, Yiwen Wang, Sung-Phil Kim

**Affiliations:** ^1^Department of Biomedical Engineering, Hanyang University, 222 Wangsimni-ro, Seongdong-gu, Seoul 133-791, Republic of Korea; ^2^School of Electrical & Computer Engineering, University of Oklahoma, Norman, OK 73019-1023, USA; ^3^Qiushi Academy of Advanced Studies, Zhejiang University, Hangzhou 310027, China; ^4^Research and Business Foundation, Korea University, Seoul 136-713, Republic of Korea

Neuroengineering is an emerging discipline in the field of medical and biological engineering with the aim of understanding, modulating, enhancing, or repairing neuronal systems, which are obviously the most complex systems of the human body. During the last decade or so, neuroengineering has been growing rapidly and expanded its applications from interpreting and processing neuronal signals to interfacing the neural systems with external devices to restore lost functions. Despite its short history, neuroengineering has now become one of the most important topics in the current biomedical engineering research. As in other interdisciplinary fields, computational methods have played key roles in the development of neuroengineering. 

Considering the aforementioned trends, it seems natural that neuroengineering is selected as the theme of this special issue. This special issue includes eleven high-quality, peer-reviewed articles that might provide researchers in the field of neuroscience, engineering, psychology, and computational sciences with the current state-of-the-art knowledge of this emerging interdisciplinary research field. 

The paper “*Trial-by-trial adaptation of movements during mental practice under force field*” by M. N. Anwar and S. H. Khan studied how motor imagery influences trial-to-trial learning in a robot based adaptation task. The results showed that reaching movements performed with motor imagery have relatively a more focused generalization pattern and a higher learning rate in training direction. 

The paper “*Evaluation of EEG features in decoding individual finger movements from one hand*” by R. Xiao and L. Ding investigates the existence of a broadband feature in EEG to discriminate individual fingers from one hand, with the significantly higher average decoding accuracy than guess level by the spectral principal component analysis (PCA). 

 The paper “*Coercively adjusted auto regression model for forecasting in epilepsy EEG*” by S.-H. Kim et al. proposes a coercively adjusted auto regression (CA-AR) method to forecast future values from a multivariate epilepsy EEG time series with higher accuracy and improved computational efficiency. 

The paper “*A mixed L2 norm regularized HRF estimation method for rapid event-related fMRI experiments*” by Y. Lei et al. presents a new regularization framework to identify trial-specific BOLD responses in extremely rapid event-related fMRI experiments, where BOLD responses are heavily overlapped from adjacent trials. It is demonstrated that the technique significantly improves the classification accuracy in decoding brain tasks in a rapid four-category object classification experiment. 

The paper “*Development of the complex general linear model in the Fourier domain: application to fMRI multiple input-output evoked responses on single subjects*” by D. E. Rio et al. describes a statistical time series analysis in the Fourier domain based on a linear time invariant model to process multivariate data from fMRI experiments, particularly for single subjects. 

The paper “*Continuous- and discrete-time stimulus sequences for high stimulus rate paradigm in evoked potential studies*” by T. Wang et al. suggests using continuous-time stimulus sequences for the high stimulus rate paradigm to obtain auditory evoked potentials (AEPs) from electroencephalography (EEG). Both the analytic results and simulation experiments revealed the advantages of using the continuous-time sequences over traditional discrete-time sequences in terms of the reliability of reconstructed AEPs. 

The review paper “*A review on the computational methods for emotional state estimation from the human EEG*” by M.-K. Kim et al. summarizes the state-of-the-art computational methods used to extract features for emotional states from EEG signals and to classify those features into one of many emotional states. The readers of the journal would find this review helpful for understanding how mathematical methods are employed for affective computing based on EEG. 

The paper “*Information analysis on neural tuning in dorsal premotor cortex for reaching and grasping*” by Y. Cao et al. investigates the contribution of the dorsal premotor cortex (PMd) in reaching and grasping experiments with monkeys. It is identified that, within PMd, there are neurons that only engage in reaching, neurons that only engage in grasping, and neurons that engage in both. This phenomenon is further validated in a decoding experiment using different sets/subsets of neurons in PMd. 

The paper “*Modiolus-hugging intracochlear electrode array with shape memory alloy*” by K. S. Min et al. presents a novel intracochlear electrode which can deliver a more efficient electrical stimulation in human cochlea to aid people with a severe hearing loss. They propose to use a shape-memory-alloy embedded electrode to reduce the distance between the electrode and the target cells. 

The paper “*Corticomuscular coherence analysis on hand movement distinction for active rehabilitation*” by X. Lou et al. evaluated corticomuscular coherence (CMC) between EEG and EMG signals recorded during voluntary hand opening tasks and found that the CMC values can detect voluntary hand opening with high accuracy. Their results suggest that CMC analysis can be a promising tool for active hand rehabilitation of patients with stroke. 

The paper “*A sound processor for cochlear implant using a simple dual path nonlinear model of basilar membrane*” by K. H. Kim et al. proposed a new active nonlinear model of the frequency response of the basilar membrane in biological cochlea called the simple dual path nonlinear (SDPN) model and a novel sound processing strategy for cochlear implants (CIs) based upon this model. 

